# Phylogenetics of a Fungal Invasion: Origins and Widespread Dispersal of White-Nose Syndrome

**DOI:** 10.1128/mBio.01941-17

**Published:** 2017-12-12

**Authors:** Kevin P. Drees, Jeffrey M. Lorch, Sebastien J. Puechmaille, Katy L. Parise, Gudrun Wibbelt, Joseph R. Hoyt, Keping Sun, Ariunbold Jargalsaikhan, Munkhnast Dalannast, Jonathan M. Palmer, Daniel L. Lindner, A. Marm Kilpatrick, Talima Pearson, Paul S. Keim, David S. Blehert, Jeffrey T. Foster

**Affiliations:** a Pathogen and Microbiome Institute, Northern Arizona University, Flagstaff, Arizona, USA; b Department of Molecular, Cellular and Biomedical Sciences, University of New Hampshire, Durham, New Hampshire, USA; c U.S. Geological Survey, National Wildlife Health Center, Madison, Wisconsin, USA; d School of Biological and Environmental Sciences, University College Dublin, Belfield, Dublin, Ireland; e Leibniz Institute for Zoo and Wildlife Research, Berlin, Germany; f Department of Ecology and Evolutionary Biology, University of California, Santa Cruz, California, USA; g Urban and Environmental Science College, Changchun Normal University, Changchun, People’s Republic of China.; h Department of Biology, School of Mathematics and Natural Sciences, The Mongolian National University of Education, Ulaanbaatar, Mongolia; i Bats Research Center of Mongolia, Ulaanbaatar, Mongolia; j U.S. Forest Service, Northern Research Station, Center for Forest Mycology Research, Madison, Wisconsin, USA; Duke University

**Keywords:** Chiroptera, *Pseudogymnoascus destructans*, emerging infectious disease, epizootic, microsatellite, whole-genome sequencing, wildlife

## Abstract

Globalization has facilitated the worldwide movement and introduction of pathogens, but epizoological reconstructions of these invasions are often hindered by limited sampling and insufficient genetic resolution among isolates. *Pseudogymnoascus destructans*, a fungal pathogen causing the epizootic of white-nose syndrome in North American bats, has exhibited few genetic polymorphisms in previous studies, presenting challenges for both epizoological tracking of the spread of this fungus and for determining its evolutionary history. We used single nucleotide polymorphisms (SNPs) from whole-genome sequencing and microsatellites to construct high-resolution phylogenies of *P. destructans*. Shallow genetic diversity and the lack of geographic structuring among North American isolates support a recent introduction followed by expansion via clonal reproduction across the epizootic zone. Moreover, the genetic relationships of isolates within North America suggest widespread mixing and long-distance movement of the fungus. Genetic diversity among isolates of *P. destructans* from Europe was substantially higher than in those from North America. However, genetic distance between the North American isolates and any given European isolate was similar to the distance between the individual European isolates. In contrast, the isolates we examined from Asia were highly divergent from both European and North American isolates. Although the definitive source for introduction of the North American population has not been conclusively identified, our data support the origin of the North American invasion by *P. destructans* from Europe rather than Asia.

## IMPORTANCE

This phylogenetic study of the bat white-nose syndrome agent, *P. destructans*, uses genomics to elucidate evolutionary relationships among populations of the fungal pathogen to understand the epizoology of this biological invasion. We analyze hypervariable and abundant genetic characters (microsatellites and genomic SNPs, respectively) to reveal previously uncharacterized diversity among populations of the pathogen from North America and Eurasia. We present new evidence supporting recent introduction of the fungus to North America from a diverse Eurasian population, with limited increase in genetic variation in North America since that introduction.

## INTRODUCTION

Fungal diseases are emerging as major threats to ecosystem integrity at local, regional, and global levels (1). For example, introduced fungal phytopathogens such as *Cryphonectria parasitica* (agent of chestnut blight) and *Cronartium ribicola* (agent of white pine blister rust) have caused dramatic declines in foundation tree species of American forests, with severe implications for regional forest structure and food webs (2, 3). Several fungal pathogens of vertebrates have also recently emerged and are of major conservation concern for wildlife, including *Batrachochytrium dendrobatidis* and *Batrachochytrium salamandrivorans* (agents of amphibian chytridiomycosis), as well as *Ophidiomyces ophiodiicola* (agent of snake fungal disease) (4–6). In particular, the ongoing chytridiomycosis panzootic ranks among the most devastating wildlife diseases due to its broad host range, high virulence, and rapid worldwide invasion (7). In 2006, another severe fungal epizootic, white-nose syndrome (WNS), emerged among hibernating bats in upstate New York in the United States. This disease was named for the white growth of the psychrophilic fungus *Pseudogymnoascus destructans* on the skin, especially muzzles, of infected bats (8). The pathogen has colonized nearly all surveyed bat hibernacula in the eastern United States and Canada over the last 10 years (9, 10). Millions of bats have died as a result of this disease, and several species are threatened with extinction, including *Myotis septentrionalis* (northern long-eared bat), the once plentiful *Myotis lucifugus* (little brown bat), and the endangered *Myotis sodalis* (Indiana bat) (11, 12). Bats provide widespread insect suppression services to natural and agricultural ecosystems and are primary suppliers of nutrients to unique cave ecosystems through deposition of guano (13). Thus, this catastrophic reduction in the bat populations of North America will likely have pervasive ecologic repercussions.

Rachowicz et al. (14) proposed the novel and endemic pathogen hypothesis for the origin of *B. dendrobatidis* and emphasized differences in management and research priorities for pathogens of different origins. A novel pathogen population would be expected to have limited diversity compared to its source population, reflecting a recent introduction and demographic bottleneck of the introduced organism (14, 15). Conversely, a pathogen with greater diversity implies a longer natural history in an area and supports the endemic pathogen hypothesis. Many bacterial, viral, parasitic, and fungal diseases of epizoological importance have expanded clonally (16), and genetic analyses of *P. destructans* have been consistent with this pattern.

*Pseudogymnoascus destructans* primarily reproduces asexually, producing abundant haploid conidia (17, 18). Although it is theoretically possible for *P. destructans* to reproduce sexually via a heterothallic mating system, this has not yet been observed, and only one of two required mating types has to date been detected in the genomes of North American fungal isolates (19). Previous studies of the origin and spread of WNS revealed little or no genetic diversity in North American isolates of *P. destructans* (20, 21). However, these previous studies were limited by low discriminatory power. Only eight loci were sequenced, and they did not exhibit sufficient polymorphisms to identify the exact source population or to distinguish among isolates of a recently emerged pathogen during an infectious disease outbreak (22).

In contrast, other genetic markers such as microsatellites (23) and whole-genome single nucleotide polymorphism (SNP) genotyping (24) use many loci from throughout the genome to increase the resolution of genotypes. The high variability of microsatellite loci leads to more heterogeneity than point mutations in genes, often allowing for discrimination between closely related or recently diverged isolates. Furthermore, the hallmark of whole-genome SNP genotyping is the large number of characters (ranging from hundreds to hundreds of thousands of SNP loci in a typical analysis) that can be used to construct a robust phylogeny of clonal organisms (22).

We report the results of a high-resolution phylogenetic analysis of *P. destructans* using both microsatellite and whole-genome SNP loci to complement the benefits and minimize the drawbacks of each approach. Our analysis includes isolates from throughout the WNS epizootic zone in North America that were collected from 2008 to 2014. We also included isolates from Europe, Mongolia, and China (where *P. destructans* was recently found to be widely distributed [25]) to more thoroughly investigate the genetic diversity of putative origin populations of the North American population.

## RESULTS

### SNP phylogeny.

**FIG 1:**
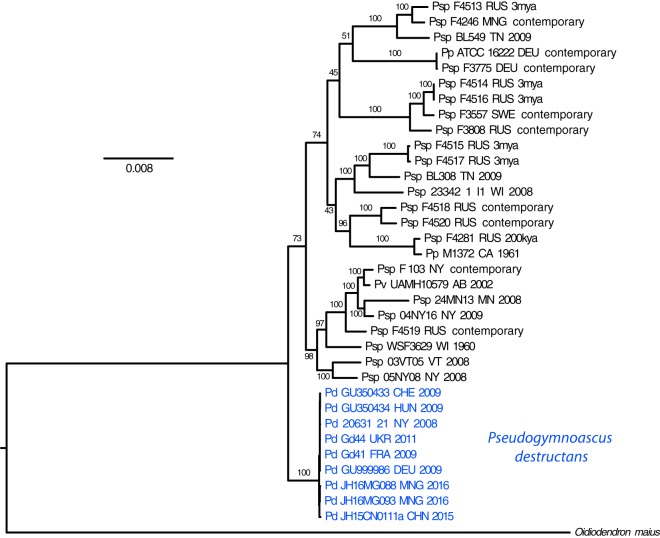
Maximum likelihood phylogenetic tree of the genus *Pseudogymnoascus* (previously *Geomyces*), based upon 4,757 SNPs (of which 2,479 are synapomorphic). Branches are labeled with bootstrap support values. *Oidiodendron maius* serves as outgroup. *Pseudogymnoascus destructans* is a sister clade to other members of the genus. Psp, *Pseudogymnoascus* sp.; Pd, *P. destructans*; Pp, *P. pannorum*; Pv, *P. verrucosus*. Additional data for each sample include isolate name, country/state of origin, and date of collection (estimated as preservation date for samples taken from permafrost). Samples without a precisely known collection date are indicated as “contemporary.”

**FIG 2:**
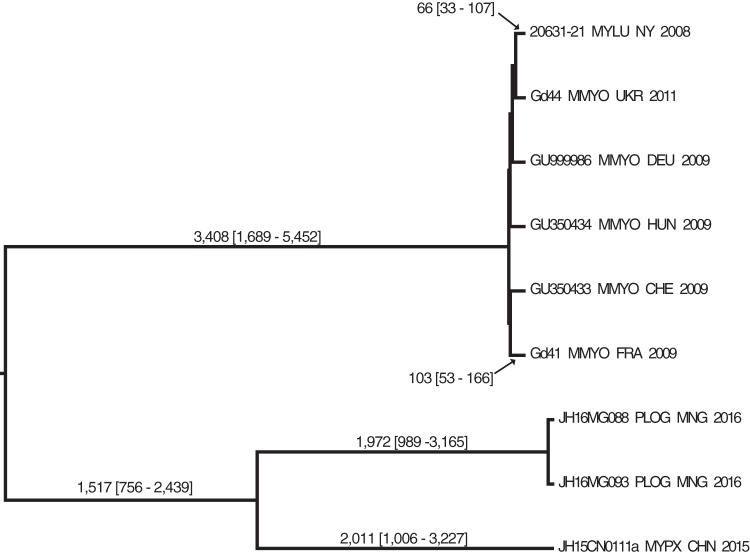
Maximum clade credibility tree for isolates of *Pseudogymnoascus destructans* from North America (*n =* 1), Europe (*n =* 5), and Asia (*n =* 3) based upon 37,752 core SNP sites (of which 22,594 are synapomorphic). All nodes had 100% posterior probabilities. Nodes are labeled with time (years before present), followed by the 95% high-probability density interval in brackets; dates on all shorter branches are not shown. North American isolates are nearly indistinguishable at these SNP loci, and so were represented in this tree by a single isolate (20631-21). The tree is rooted on the Chinese isolate based upon the Fig. 1 phylogeny that shows other *Pseudogymnoascus* spp. as a sister clade to *P. destructans*. Data for each sample include isolate name, bat host species code, country/state of origin, and date of collection.

Illumina paired-end whole-genome shotgun sequences were obtained for 26 isolates of *P. destructans* from the North American WNS epizootic zone from the years 2008 to 2014, 5 isolates from Europe, and 3 isolates from Asia. These reads, as well as publicly available *P. destructans* and near-relative *Pseudogymnoascus* and *Geomyces* species assemblies, were aligned to a reference genome from *P. destructans* 20631-21 (NCBI accession no. GCA_001641265.1) (26). *Oidiodendron maius* Zn (*Ascomycota*, *Leotiomycetes*) was used as an outgroup based on our phylogenetic analyses of fungi closely related to *Pseudogymnoascus* (data not shown). Descriptive statistics of the alignments, as well as NCBI accession numbers of all samples analyzed, are displayed in Table 1. Duplicated/repeat regions accounted for 43.25% of the genome and were excluded from our SNP-based analyses. Only ~0.1% of the reference genome was orthologous in all samples, resulting in 4,757 SNP loci, of which 2,479 were synapomorphic (i.e., shared between one or more samples and useful to define clades). Maximum parsimony analysis resulted in four trees with a low rescaled consistency (RC) index (RC of 0.4704), indicating a high degree of homoplasy that one would expect from a data set representing a long evolutionary history (data not shown). Maximum likelihood analysis (Fig. 1) indicated strong bootstrap support for a single *P. destructans* clade that is sister to other all currently recognized members of the genus *Pseudogymnoascus*, including ancient samples from Russian permafrost. Moreover, in this analysis, isolate JH15CN0111a from China is basal to *P. destructans* isolates from Mongolia, Europe, and North America, allowing us to root the *P. destructans* phylogeny (Fig. 2).

**TABLE 1:** Whole-genome sequences aligned to the reference genome of *Pseudogymnoascus destructans* (NCBI accession no. GCA_001641265.1)

Isolate ID no.	Species^a^	Source^b^	Location^c^	Date	Read length (bp)	Avg read depth	% of core genome covered^d^	Accession no.
20631-21	*P. destructans*	*M. lucifugus*	NY	Feb 2008	NA^e^	NA	100.00	GCA_001641265.1
GU999986	*P. destructans*	*M. myotis*	DEU	Mar 2009	2 × 10^1^	155.83	98.73	SRR6011467
GU350433	*P. destructans*	*M. myotis*	CHE	Apr 2009	2 × 10^1^	187.97	98.93	SRR6011468
GU350434	*P. destructans*	*M. myotis*	HUN	Mar 2009	2 × 10^1^	169.29	99.00	SRR6011465
20693-1	*P. destructans*	*M. lucifugus*	MA	Mar 2008	2 × 10^1^	206.39	99.79	SRR6011466
22429-8	*P. destructans*	*M. septentrionalis*	WV	Jan 2009	2 × 10^1^	208.62	99.81	SRR6011471
22971-3	*P. destructans*	*M. lucifugus*	ON	Mar 2010	2 × 10^1^	123.24	99.73	SRR6011472
22504-1	*P. destructans*	*M. lucifugus*	PA	Mar 2009	2 × 10^1^	192.73	99.67	SRR6011469
22442-2	*P. destructans*	*M. lucifugus*	NJ	Feb 2009	2 × 10^1^	200.79	99.71	SRR6011470
22948-1	*P. destructans*	*M. septentrionalis*	TN	Mar 2010	2 × 10^1^	180.47	99.49	SRR6011473
20674-9	*P. destructans*	*M. septentrionalis*	VT	Mar 2008	2 × 98	100.25	99.46	SRR6011474
20682-10	*P. destructans*	*M. septentrionalis*	MA	Mar 2008	2 × 10^1^	156.00	99.11	SRR6011477
22004-1	*P. destructans*	*M. lucifugus*	CT	Apr 2008	2 × 10^1^	213.78	99.20	SRR6011478
22426-2	*P. destructans*	*M. lucifugus*	CT	Jan 2009	2 × 10^1^	170.49	99.42	SRR6011475
22469-1	*P. destructans*	*P. subflavus*	VA	Mar 2009	2 × 10^1^	167.09	99.42	SRR6011476
22480-1	*P. destructans*	*E. fuscus*	NY	Mar 2009	2 × 98	85.85	99.43	SRR6011481
22884-4W	*P. destructans*	*M. lucifugus*	VT	Jan 2010	2 × 10^1^	141.88	99.43	SRR6011482
22930-2	*P. destructans*	*P. subflavus*	TN	Feb 2010	2 × 10^1^	82.98	99.03	SRR6011479
22949-4	*P. destructans*	*M. septentrionalis*	MD	Mar 2010	2 × 10^3^	211.59	99.54	SRR6011480
22972-2W	*P. destructans*	*M. lucifugus*	ON	Mar 2010	2 × 10^1^	82.48	99.03	SRR6011483
22997-1	*P. destructans*	*M. septentrionalis*	TN	Apr 2010	2 × 10^1^	271.20	99.97	SRR6011484
23414-1W	*P. destructans*	*M. lucifugus*	IN	Jan 2011	2 × 98	120.00	99.59	SRR6011493
23434-1W	*P. destructans*	*M. lucifugus*	IN	Jan 2011	2 × 10^1^	255.07	99.96	SRR6011492
23444-1	*P. destructans*	*M. lucifugus*	TN	Feb 2011	2 × 98	96.67	99.49	SRR6011491
23455-1	*P. destructans*	*M. lucifugus*	VA	Feb 2011	2 × 10^1^	163.17	99.70	SRR6011490
Gd41	*P. destructans*	*M. myotis*	FRA	Mar 2009	2 × 251	89.04	99.31	SRR6011497
Gd44	*P. destructans*	*M. myotis*	UKR	Feb 2011	2 × 301	92.68	98.71	SRR6011496
23874-1	*P. destructans*	*M. lucifugus*	ME	Dec 2011	2 × 10^1^	200.76	99.97	SRR6011495
23877-1	*P. destructans*	*M. septentrionalis*	DE	Mar 2012	2 × 10^1^	177.32	99.97	SRR6011494
23897-2	*P. destructans*	*P. subflavus*	MO	Mar 2012	2 × 251	119.80	99.93	SRR6011489
W41203	*P. destructans*	Sub	NB	Apr 2012	2 × 251	74.31	99.54	SRR6011488
JH15CN0111a	*P. destructans*	*M. petax*	CHN	Mar 2015	2 × 150	74.31	96.78	SRR6011485
JH16MG088	*P. destructans*	*P. ognevi*	MNG	2016	2 × 150	78.84	97.31	SRR6011486
JH16MG093	*P. destructans*	*P. ognevi*	MNG	2016	2 × 150	15.28	53.10	SRR6011487
03VT05	*Pseudogymnoascus* sp.	Sed	VT	2008	NA	NA	76.63	GCA_001662645.1
UAMH10579	*P. verrucosus*	Peat	AB	2002	NA	NA	76.92	GCA_01662655.1
05NY08	*Pseudogymnoascus* sp.	Sed	NY	2008	NA	NA	76.68	GCA_001662605.1
23342-1-I1	*Pseudogymnoascus* sp.	*P. subflavus*	WI	2008	NA	NA	61.20	GCA_001662575.1
24MN13	*Pseudogymnoascus* sp.	Sed	MN	2008	NA	NA	69.28	GCA_001662595.1
WSF3629	*Pseudogymnoascus* sp.	Peat	WI	1960	NA	NA	77.02	GCA_001662585.1
F-103	*Pseudogymnoascus* sp.	Soil	NY	Contemporary	NA	NA	76.76	GCA_000750895.1
F-3557	*Pseudogymnoascus* sp.	Pine post	SWE	Contemporary	NA	NA	52.04	GCA_000750665.1
F-3775	*Pseudogymnoascus* sp.	Soil	DEU	Contemporary	NA	NA	50.60	GCA_000750715.1
F-3808	*Pseudogymnoascus* sp.	*M. glareolus*	RUS	Contemporary	NA	NA	52.51	GCA_000750675.1
F-4246	*Pseudogymnoascus* sp.	Sed	MNG	Contemporary	NA	NA	53.77	GCA_000750735.1
F-4281	*Pseudogymnoascus* sp.	Cryopeg	RUS	0.12–0.2 mya^f^	NA	NA	57.09	GCA_000750745.1
F-4513	*Pseudogymnoascus* sp.	Permafrost	RUS	1.8–3.0 mya	NA	NA	54.09	GCA_000750755.1
F-4514	*Pseudogymnoascus* sp.	Permafrost	RUS	1.8–3.0 mya	NA	NA	51.80	GCA_000750795.1
F-4515	*Pseudogymnoascus* sp.	Permafrost	RUS	1.8–3.0 mya	NA	NA	61.24	GCA_000750805.1
F-4516	*Pseudogymnoascus* sp.	Permafrost	RUS	1.8–3.0 mya	NA	NA	53.86	GCA_000750815.1
F-4517	*Pseudogymnoascus* sp.	Permafrost	RUS	1.8–3.0 mya	NA	NA	60.86	GCA_000750875.1
F-4518	*Pseudogymnoascus* sp.	Soil	RUS	Contemporary	NA	NA	67.47	GCA_000750925.1
F-4519	*Pseudogymnoascus* sp.	Soil	RUS	Contemporary	NA	NA	77.04	GCA_000750935.1
F-4520	*Pseudogymnoascus* sp.	Soil	RUS	Contemporary	NA	NA	67.01	GCA_000750935.1
ATCC 16222	*P. pannorum*	Soil	DEU	NA	NA	NA	58.08	GCA_001630605.1
M1372	*P. pannorum*	Soil	CA	Jun 1961	NA	NA	58.55	GCA_000497305.1
Zn	*O. maius*	NA	NA	NA	NA	NA	0.29	GCA_000827325.1

a The species shown are *Pseudogymnoascus destructans*, *Pseudogymnoascus verrucosus*, *Pseudogymnoascus* sp., *Pseudogymnoascus pannorum*, and *Oidiodendron maius*.

b The species shown are *Myotis myotis*, *Myotis lucifugus*, *Myotis septentrionalis*, *Perimyotis subflavus*, *Eptesicus fuscus*, *Myotis petax*, *Plecotus ognevi*, and *Myodes glareolus* (bank vole). Sub, hibernaculum substrate (i.e., roosting surface); Sed, hibernaculum sediment.

c NY, New York; DEU, Germany; CHE, Chechnya; HUN, Hungary; MA, Massachusetts; WV, West Virginia; ON, Ontario, Canada; PA, Pennsylvania; NJ, New Jersey; TN, Tennessee; VT, Vermont; CT, Connecticut; VA, Virginia; MD, Maryland; IN, Indiana; FRA, France; UKR, Ukraine; ME, Maine; DE, Delaware; MO, Missouri; NB, New Brunswick; CHN, China; MNG, Montenegro; AB, Alberta; WI, Wisconsin; MN, Minnesota; SWE, Sweden; RUS, Russia; CA, California.

d Coverage reported as percentage of core genome (i.e., portion of genome shared by all samples that is not in a duplicated region) that passed 10× minimum depth of coverage and 90% of read agreement filters.

e NA, not available.

f mya, million years ago.

As previously determined by multilocus sequence typing (MLST) (27), members of the genus *Pseudogymnoascus* are not all closely related to each other. We thus limited the sample set to just *P. destructans* and aligned only reads from this species to identify SNP loci shared specifically among *P. destructans* isolates. This resulted in the detection of 37,752 core SNP loci within the 20-Mb core genome alignment. Maximum parsimony produced one tree with an RC of 0.7729 (data not shown). Maximum likelihood analysis (Fig. 2) distinguishes three well-supported clades among the *P. destructans* isolates: China, Mongolia, and Europe. The North American isolates are nearly identical in this tree and are therefore represented as a group by the type isolate 20631-21, obtained in New York in 2008. This phylogeny clearly shows the North American clade of *P. destructans* as a member of a greater European clade and distinct from the eastern and central Asian isolates. The tree also shows that North American *P. destructans* isolate is most closely related to a Ukrainian isolate (Gd44) in our sample set and least related to an isolate from France (Gd41). This is in contrast to a previous MLST study (28), which showed the opposite trend using many of the same isolates. However, low sample sizes, long branch lengths, and poor bootstrap support for the branches between members of the European clade preclude stronger conclusions about the region of Europe that gave rise to North American *P. destructans*. With our current data set, the possibility that North American *P. destructans* was introduced from Asia, however, seems unlikely.

We reduced the sample set once again to include only isolates of *P. destructans* from North America and reanalyzed the alignments for SNPs. Only 51 core SNP loci were shared among the 26 North American isolates. Maximum parsimony produced one tree with an RC of 1.00 (data not shown). Thus, all homoplasy observed among *P. destructans* phylogenies was due to the Eurasian genomes. Only three SNPs were synapomorphic. Two SNPs show differences between the type isolate 20631-21 and all other samples. A single SNP shared by samples 23414-1W and 23434-1W, both obtained in Indiana in 2011, suggest the formation of a geographically isolated lineage. Less conservative filtering steps for SNP quality and read depth and removing the requirement for a locus to be present in all genomes failed to substantially increase the number of synapomorphies. Of the 51 SNP loci, none of the mutations were in coding sequences (Table 2), but rather were from intergenic sequences, introns, or untranslated regions (UTRs) of exons.

**TABLE 2:** Annotation of core SNP loci from isolates of *P. destructans* from North America

Accession no.	Position	Type	Variant isolate(s)	Protein ID no.	Product
KV441386.1	1202839	UTR^a^	20693-1_MA_2008	OAF63413.1	NAD synthase
KV441386.1	2276958	Intergenic	22948-1_TN_2010	NA^b^	NA
KV441395.1	783753	UTR	22948-1_TN_2010	OAF58958.1	Hypothetical protein
KV441396.1	546602	Intergenic	23444-1_TN_2011	NA	NA
KV441398.1	551631	UTR	23877-1_DE_2012	OAF57976.1	Hypothetical protein
KV441399.1	235966	Intergenic	23414-1W_IN_2011, 23434-1W_IN_2011	NA	NA
KV441400.1	640730	UTR	22442-2_NJ_2009	OAF57273.1	Hypothetical protein
KV441401.1	139816	Intergenic	22948-1_TN_2010	NA	NA
KV441402.1	72139	Intergenic	23897-2_MO_2012	NA	NA
KV441402.1	226767	UTR	22948-1_TN_2010	OAF56852.1	Hypothetical protein
KV441402.1	510412	Intergenic	20693-1_MA_2008	NA	NA
KV441404.1	262446	UTR	22442-2_NJ_2009	OAF56419.1	Hypothetical protein
KV441387.1	430984	Intergenic	23874-1_ME_2012	NA	NA
KV441387.1	820735	UTR	22426-2_CT_2009	OAF62808.1	Hypothetical protein
KV441387.1	1825781	Intergenic	22948-1_TN_2010	NA	NA
KV441387.1	2082665	UTR	22442-2_NJ_2009	OAF62574.1	ESCRT-I complex subunit VPS28
KV441387.1	685734	Intergenic	22442-2_NJ_2009	NA	NA
KV441387.1	785423	Intergenic	23897-2_MO_2012	NA	NA
KV441387.1	54855	Intergenic	22971-3_ON_2010	NA	NA
KV441387.1	193542	Intergenic	22948-1_TN_2010	NA	NA
KV441387.1	253155	Intergenic	22442-2_NJ_2009	NA	NA
KV441387.1	77282	Intergenic	23455-1_VA_2011	NA	NA
KV441387.1	120236	Intergenic	23897-2_MO_2012	NA	NA
KV441388.1	775461	Intragenic	22972-2W_ON_2010	OAF61744.1	Hypothetical protein
KV441388.1	950597	Intron	23897-2_MO_2012	OAF61915.1	Hypothetical protein
KV441388.1	120202	UTR	22469-1_VA_2009	OAF62044.1	Hypothetical protein
KV441388.1	57283	UTR	20631-21_NY_2008	OAF61813.1	Hypothetical protein
KV441389.1	343287	Intron	23455-1_VA_2011	OAF61688.1	AGC/AKT protein kinase
KV441389.1	419134	Intergenic	23414-1W_IN_2011	NA	NA
KV441389.1	867065	UTR	22004-1_CT_2008	OAF61368.1	Actin cytoskeleton-regulatory complex protein end3
KV441389.1	1173430	Intergenic	22971-3_ON_2010	NA	NA
KV441389.1	1173432	Intergenic	22971-3_ON_2010	NA	NA
KV441389.1	50002	UTR	22948-1_TN_2010	OAF61394.1	Hypothetical protein
KV441390.1	643943	Intergenic	23897-2_MO_2012	NA	NA
KV441390.1	659862	UTR	22948-1_TN_2010	OAF61216.1	Hypothetical protein
KV441390.1	861423	UTR	22948-1_TN_2010	OAF60933.1	Hypothetical protein
KV441390.1	1243398	UTR	23434-1W_IN_2011	OAF60771.1	Hypothetical protein
KV441390.1	1524359	UTR	22948-1_TN_2010	OAF60872.1	Hypothetical protein
KV441391.1	151986	UTR	20693-1_MA_2008	OAF60366.1	Hypothetical protein
KV441391.1	1184294	Intergenic	22442-2_NJ_2009	NA	NA
KV441392.1	712894	Intergenic	23414-1W_IN_2011	NA	NA

a UTR, untranslated region of exon.

b NA, not available.

Isolate mating type was determined from whole-genome sequence alignments to the reference genome sequence of type isolate 20631-21, which was previously determined to mating type MAT1-1 (19). All isolates from North America in this study possessed the MAT1-1 mating type. Most Eurasian isolates did as well, although an isolate from Switzerland (GU350433) and an isolate from Mongolia (JH16MG088) were mating type MAT1-2.

### Microsatellite phylogeny.

**FIG 3:**
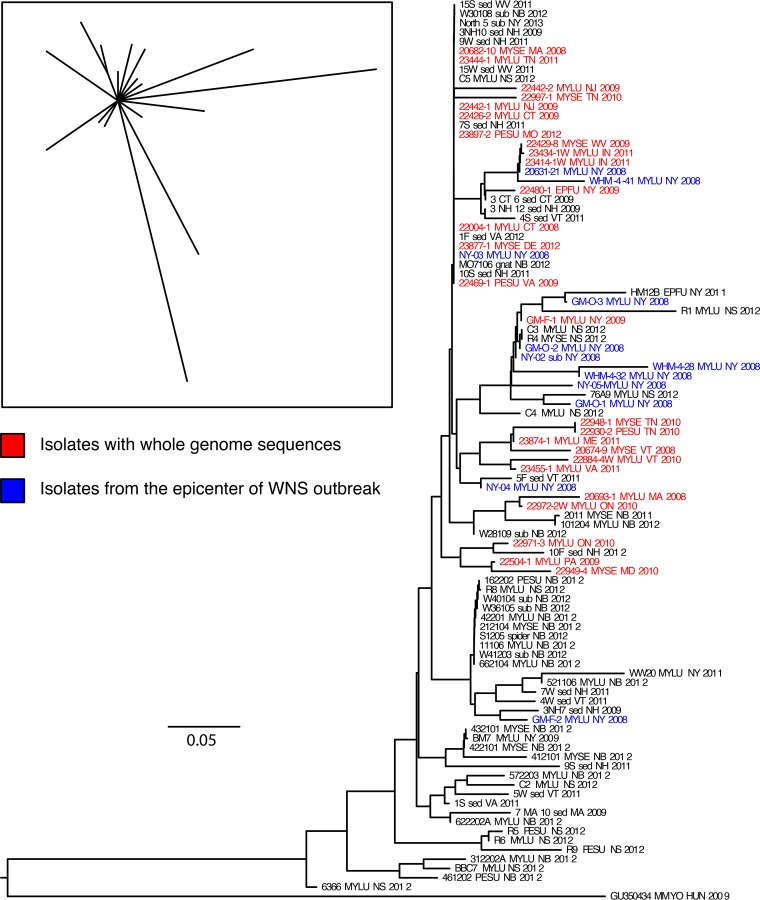
Phylogeny of 96 North American *Pseudogymnoascus destructans* isolates constructed from alleles of 23 microsatellite loci. Isolates that were included in whole-genome sequence phylogenies are colored red. The scale bar indicates genetic distance. Shown is a neighbor-joining tree of 96 North American isolates rooted with GU350434 (host, *M. myotis* [MMYO]; location, Hungary [HUN]; date, 2009). Samples from the epicenter of the WNS epizootic are colored blue. (Inset) Star-shaped maximum parsimony tree from comparisons of whole genomes, constructed from 51 SNPs, only 3 of which are synapomorphic.

Insufficient synapomorphic SNPs were discovered among the North American *P. destructans* whole-genome sequences to produce an informative phylogeny, such that analyses produced only a star-shaped phylogeny (inset in Fig. 3). Therefore, 96 North American isolates of *P. destructans*, including those analyzed by whole-genome sequencing, were genotyped with a 23-locus microsatellite panel (see Table S1 in the supplemental material). Thirty-one unique genotypes were identified, with one dominant genotype for 13 of the isolates. A phylogeny was constructed by the neighbor-joining (NJ) method from the microsatellite data (Fig. 3). These data, although based upon fewer genetic loci, contain many more samples and reveal far more genetic diversity than the SNP-based phylogeny of North American *P. destructans*. This tree supports the single clade containing isolates 23431-1W and 23434-1W from Indiana identified in the whole-genome phylogeny, but includes a number of other isolates that did not share synapomorphic SNPs with the Indiana isolates. Other clades are evident as well, but they do not seem to correspond to geographic regions or sampling dates. A Mantel test comparing microsatellite-based genetic distance to geographic distance between isolates showed no correlation (*r* = 0.016, *n =* 99 replicates, *P* = 0.37). We note, however, that despite the appearance of defined groupings throughout the tree, there was limited bootstrap support for all of the branches within this phylogeny (all bootstrap values were <70).

10.1128/mBio.01941-17.1 TABLE S1  Microsatellite panel data. Download TABLE S1, XLS file, 0.07 MB. Copyright © 2017 Drees et al. 2017 Drees et al. This content is distributed under the terms of the Creative Commons Attribution 4.0 International license.

### Population genetics.

Indices of genetic diversity and linkage were calculated for SNP genotypes. Asian samples were left out of this analysis due to the low sample size. The Simpson’s index of genetic diversity was reduced in North America (λ = 0.690, standard error [SE] = 0. 130) compared to Europe (λ = 0.800), the putative source population for *P. destructans*. Average genomic linkage, represented by standardized index of association (29), was close to zero for both populations. Among North American isolates, *ṝ*_*D*_ = 0.0585, whereas for Europe, *ṝ*_*D*_ = 0.00812. Thus, recombination does not appear to be a significant factor in either population’s genetic structure, and *P. destructans* isolates in both regions are reproducing clonally. The evenness of our sampling was not sufficient, nor did our data conform to various model assumptions to produce reliable population structure estimates (using models such as STRUCTURE and SNMF) from our SNP or microsatellite data (30).

## DISCUSSION

### Phylogeography.

The two genetic markers used in this study, SNPs and microsatellites, provide complementary information regarding the phylogeography of *P. destructans*. Whole-genome sequencing revealed three distinct populations of *P. destructans* among the isolates we tested: one consisting of European isolates that included WNS epizootic isolates from North America, another of Mongolian isolates, and a third represented by an isolate from China. The phylogeny of European isolates determined in this study with 58,707 SNP loci was consistent with that described previously using 14 SNPs in an eight-member multilocus sequence typing analysis (MLST) (28): specifically, the genetic diversity of *P. destructans* was 14% higher in Europe than in North America. In this study, we identified only 51 core SNPs among North American isolates, which clearly demonstrates the homogeneity of *P. destructans* population structure across the epizootic zone and is consistent with previous reports of clonal spread (20, 21, 28, 31, 32). North American isolates formed a monophyletic group but appear to be representative of diversity from Europe (i.e., the branch lengths between the European samples were comparable between the European and North American isolates). This, as well as the 99.995% reduction in core SNP loci when non-American isolates are removed, represents a loss of genetic diversity consistent with previous conclusions of a recent introduction of a novel pathogen (33, 34). Moreover, star-shaped phylogenies containing few synapomorphies are typical in recent pathogen introductions (35–37), with lack of rapid diversification into distinct lineages likely representing the norm rather than the rule. A recent report on the spread of WNS also identified few mutations of *P. destructans* upon introduction to North America (32). A strict interpretation of the dates in Fig. 2 would suggest that all *P. destructans* strains in North America and Europe diverged from each other within the past ~100 years and from the ancestor to Asian isolates roughly 3,400 years ago. While these estimates are plausible, we believe a more accurate estimation will require increased sampling of isolates from Eurasian populations. Perhaps analyses using *P. destructans* genomes from samples preserved long ago, as we did with *Pseudogymnoascus* sp. genomes from ancient Russian permafrost samples in Fig. 1, or detection of WNS-induced bat die-offs in Eurasia from the fossil record will enable more accurate dating. Given that *P. destructans* has likely been a bat pathogen for millions of years (73), the lack of mortality in European bats, considerable genetic diversity in microsatellite loci, and the broad distribution of the fungus across the palearctic—all changes that we envision taking millennia to evolve—it is likely that *P. destructans* is much older than a few thousand years old. Clearly more detailed molecular dating analyses are needed to resolve the question of the emergence and diversification of this species.

The microsatellite-based phylogeny containing more isolates illustrates some additional epizoological features with relevance to pathogen invasion and establishment. What is most striking about these data is the large amount of genetic diversity revealed among the North American isolates. Earlier studies indicated that 73 isolates of *P. destructans* from New York, Vermont, Pennsylvania, Ohio, West Virginia, North Carolina, Ontario, New Brunswick, Nova Scotia, and Prince Edward Island were genetically identical via MLST (20, 21, 31). Genotyping of these samples plus 78 others from eastern Canada and the United States resulted in a single genotype in 109 samples, plus two singleton genotypes from New Brunswick and one from Ontario. In contrast, microsatellite loci from our study defined 31 different genotypes among the isolates from North America. Another hallmark of these data is the lack of geographic or temporal correspondence between clades evident in the tree. For example, the Fig. 3 phylogeny shows that isolates collected from New York in 2008, at the beginning of the WNS outbreak, are distributed throughout the tree. Some isolates from the same sampling location and date (e.g., the Williams Hotel Mine) occur in different clades, indicating relatively high genetic diversity from this one region compared to other regions in North America. Unfortunately, these data do not indicate whether this diversity was present in the founding population or arose soon after introduction, a common problem with population genetic analyses of pathogens (38). New Brunswick and Nova Scotia isolates also occur in many clades associated with isolates from throughout the epizootic zone. A possible exception to the lack of geographic structuring we observed are several basal clades containing only northeastern Canadian isolates or isolates from New England and New York early in the outbreak. This region is relatively isolated and may represent an area that received *P. destructans* isolates geographically spreading from New England early in the outbreak and has had time since then to diversify genetically. Intense sampling in this region may also be contributing sampling bias to this observation, however.

### Clonality.

Questions regarding clonal expansion of *P. destructans* in North America can be answered more thoroughly by this study due to the large number of characters used in this data set. The strongest evidence for clonal reproduction of an ascomycete fungus is the lack of both mating types in a population, whereas an even distribution of mating types in a population would suggest the prevalence of sexual recombination. In a previous study (19), 5 out of 23 (22%) of isolates from Eastern Europe possessed the *MAT1-2* locus. With the addition of our samples from Eurasia, the proportion of MAT1-2 to MAT1-1 mating types is 7 out of 30 isolates (23%). In contrast, all 26 of the isolates from North America in this study possessed the MAT1-1 mating type. Additional analysis of *P. destructans* mating types in an eastern European population showed a more equal distribution of mating types (58.6% MAT1-1 and 41.3% MAT1-2; *n =* 41) (39). The absence of the MAT1-2 mating type in North American *P. destructans* is evidence of both asexual reproduction and likely a recent genetic bottleneck.

The overrepresentation of widespread and persistent genotypes is also evidence of clonal reproduction as opposed to sexual reproduction (40), as was indicated by both SNP and microsatellite analyses in this study. Based upon SNP genotyping, 8 of the 26 isolates sequenced (31%) were found to be clonal, ranging from New England to Tennessee and sampled between 2008 and 2010. Similarly, two microsatellite genotypes account for 15 and 6 samples, respectively, out of a total of 55 North American isolates for which we obtained complete microsatellite genotypes (38%). Quantitatively, the Simpson’s index of genetic diversity was slightly reduced in North America, although indexes of genetic diversity are highly dependent upon sample size, despite rarefaction, and the number of isolates from Europe compared to the number of isolates in North America analyzed in this study is very small. Average genomic linkage as measured by the standardized index of association indicates that both the European and North American populations of *P. destructans* are at linkage equilibrium, suggesting both populations reproduce primarily as clones.

### Introduction of *P. destructans* to North America.

Warnecke et al. (41) hypothesized that *P. destructans* was a novel pathogen introduced to North America from Europe based upon evidence that North American little brown bats developed WNS when inoculated with a European isolate of *P. destructans*. Their findings were further strengthened by Leopardi et al. (28), who demonstrated genetic similarity, based on MLST, between the North American and some European fungal populations, indicating the likely source population for this introduction to be from Europe. Subsequently, *P. destructans* has been isolated from bats throughout Europe (39, 42–46) and can also cause histopathological lesions consistent with WNS in European species of bats (47, 48). However, widespread mortality of bats from WNS has not been documented on the European continent (49). This suggests European bat species may have developed resistance or tolerance to infection by *P. destructans*, presumably due to coevolution between hosts and the fungal pathogen (41, 50). In contrast, the high mortality of North American bats infected with *P. destructans* is consistent with exposure of naive host species to a pathogen preadapted to similar hosts and environmental conditions.

Our work supports the hypothesis that *P. destructans* is a novel pathogen recently introduced to North America by demonstrating the relationship of North American isolates of the fungus to a population dominated by European isolates but genetically distant from isolates from Asia. Using high-resolution genetic markers, we document a loss of genetic diversity in epizootic *P. destructans* consistent with a recent introduction of this pathogen to North America.

### Similarities to other fungal invasions.

*Pseudogymnoascus destructans* follows a pattern of emergence and spread similar to other invasive fungal pathogens. In the case of *Cryphonectria parasitica*, the causative agent of chestnut blight (38), high genetic diversity in putative source populations coupled with a significant decrease in allelic richness in microsatellite and other multilocus genotypes among North American isolates suggested a founder effect associated with the introduction of the organism from Asia. North American isolates of *C. parasitica* were as genetically distant from Asian populations as the Asian populations were from each other. Analysis of population structure could narrow the source population down to a broad region (i.e., Honshu, Japan), but required the admixture of an unsampled population significantly different from the other Asian samples. A more detailed genomic analysis of an invading fungus was conducted with isolates of *B. dendrobatidis* originating from multiple continents and revealed a considerably more complex phylogeny of that fungus than was previously determined. A virulent panzootic strain apparently emerged and rapidly spread across the globe, yet the original source population of the lineage remains ambiguous despite a comprehensive sampling effort and thorough sequencing analyses (51). Consistent with a pattern also seen in our study, haplotypes from *B. dendrobatidis* did not correlate well with host or geography, suggesting other drivers of differentiation. Additionally, phylogenies constructed from different types of genetic markers yielded different, sometimes contradictory results for clones of the potato blight agent, *Phytophthora infestans* (52). A common thread in all of these case studies is that more genetic information lends complexity, but not necessarily clarity, to the natural history of an invading fungal pathogen. In terms of the natural history of *P. destructans*, it will require extensive sampling in Europe to find the population that was introduced to North America.

Using two high-resolution genetic characters—SNPs within whole-genome sequences and microsatellites—we have demonstrated that isolates of *P. destructans* in North America form a single clade much more closely related to isolates from Europe than to genetically distant populations sampled in Asia. The North American isolates represent a loss of genetic diversity in comparison to isolates from Europe, adding further support to the hypothesis that *P. destructans* was recently introduced to North America from Europe (28). Future sampling in Europe will be needed to more precisely define the origin of *P. destructans*, although the substantial diversity of *P. destructans* in Europe, coupled with results from other fungal invasion studies, suggests that identification of the exact source population may be challenging. Microsatellite loci were particularly suitable for distinguishing isolates from throughout the North American WNS epizootic zone, but the few synapomorphic SNPs found among these isolates could not resolve their phylogenetic relationships. However, sufficient SNPs were present to differentiate isolates of *P. destructans* from Europe, China, Mongolia, and North America. This work elevates our understanding of the origin and spread of *P. destructans* to a similar level to that achieved for *B. dendrobatidis* and *C. parasitica*, providing a framework for a global pathogen population structure that can be used for future epizoological investigations.

## MATERIALS AND METHODS

### Sampling and culturing *P. destructans*.

Skin samples from bats or swab samples of bats and substrates were cultured on Sabouraud dextrose agar or potato dextrose agar and incubated at 7 to 10°C, as previously described (42, 53).

### Whole-genome sequencing.

Genomic DNA was extracted from *P. destructans* by a variety of methods, including the OmniPrep for Fungi kit (G-Biosciences, St. Louis, MO), the DNeasy blood and tissue kit (Qiagen, Hilden, Germany) using the supplementary protocol from the manufacturer “Purification of total DNA from yeast using the DNeasy Blood and Tissue kit (DIY13 Aug-06)” as described previously (54), or a phenol-chloroform extraction. Preparation of libraries for Illumina whole-genome sequencing was based upon a previously published method (55) but was modified as sequencing technology evolved during the course of the project. In summary, approximately 1 to 10 µg of DNA sample in 200 µl Tris-EDTA buffer was sheared with a SonicMan microplate sonicator (Brooks Automation, Chelmsford, MA) to produce fragments 200 to 1,000 bp in length, with the average fragment length being 600 bp. The shearing protocol was as follows: 75 s at 0°C prechill, followed by 20 cycles of sonication for 10.0 s at full power, 75 s at 0°C lid chill, 10 s at 0°C plate chill, and 75 s at 0°C postchill. End repair, dA-tailing, adapter ligation, and indexing followed the standard Illumina protocol “Preparing samples for multiplexed paired-end sequencing” (part no. 1005361) using reagents from the NEBNext DNA library prep master mix set for Illumina (New England Biolabs, Ipswich, MA) or the KAPA high-throughput library preparation kit “with bead” (Kapa Biosystems, Wilmington, MA). Libraries were indexed with the Illumina multiplexing sample preparation oligonucleotide kit 6-bp indices (Illumina, San Diego, CA) or custom 8-bp indices for higher index read discrimination (56). Size selection of 600-bp fragments was accomplished by excision from 2% agarose gels followed by purification with a QIAquick gel purification kit (Qiagen, Hilden, Germany), E-Gel SizeSelect gel (Life Technologies, Inc., Grand Island, NY), or Agencourt AMPure XP SPRI beads (Beckman Coulter, Inc., Indianapolis, IN) using the Kapa Biosystems “High-throughput NGS library preparation technical guide, Illumina platforms” (57). Products from each step of the library preparation were purified with either Agencourt AMPure XP SPRI beads using the same protocol or the QIAquick 96 PCR purification kit (Qiagen, Hilden, Germany). Libraries were quantified via quantitative PCR with a KAPA library quantification kit (Kapa Biosystems, Wilmington, MA) for the ABI Prism 9600 real-time PCR system (Life Technologies, Inc., Grand Island, NY). Prior to loading on the sequencer, the library fragment size distribution was qualitatively confirmed with an Agilent DNA high-sensitivity kit for the 2100 Bioanalyzer (Agilent, Santa Clara, CA). Individual libraries were run on an Illumina GAIIx sequencer (V2 chemistry) or an Illumina MiSeq (V3 chemistry) to produce 100-bp paired-end reads or 250-bp paired-end reads, respectively. Libraries sequenced on a HiSeq 2000 were multiplexed with a maximum of five libraries per lane to produce 150-bp paired-end reads with V3 chemistry.

### Whole-genome sequence analysis.

The sequence from resequencing and assembly of the genome of *P. destructans* 20631-21, collected in New York in 2008 (26) (NCBI GenBank assembly accession no. GCA_001641265.1), was used as a reference for SNP discovery using the Northern Arizona SNP Pipeline (NASP) version 1.0.2 (58). With NASP, Illumina paired-end reads were aligned to the reference genome with the Burrows-Wheeler Aligner (59) version 0.7.5a mem algorithm. SNPs were detected in the alignments with the Unified Genotyper of the Genome Analysis Toolkit (60) build 2.5-2-gf57256b following recommended best practices (61, 62). Duplicated regions of the reference genome, including repeat regions and multiple gene copies, were determined by aligning the reference sequence to itself using the nucmer algorithm of MUMmer version 3.23 (63). SNPs that fell within these duplicate regions were excluded from further analysis to avoid false SNP calls due to ambiguous read alignment. SNP loci were also excluded if at least one sample lacked a base call at an SNP locus), had read coverage of less than 10× in at least one sample, and less than 90% of the reads agreed with the SNP call. Default settings for each software package were used unless otherwise noted. Mating type was determined from the prevalence of reads mapping to the *MAT1-1* mating type locus in read alignments to the reference sequence (NCBI GenBank accession no. KV441390.1, bases 187500 to 188500). SNPs were annotated with SnpEff version 4.3q (64).

### Microsatellite genotyping.

DNA samples from *P. destructans* were tested with the 23-locus multilocus variable number tandem-repeat analysis (MLVA) panel described by Drees et al. (65). Briefly, we identified 2- to 6-bp repeats in the genome sequence of *P. destructans* type strain 20631-21 (GenBank accession no. GL573169 to GL575015). We then tested 127 primer pairs for microsatellites with length polymorphisms using a diverse set of DNA extracts from 39 isolates of *P. destructans* collected from North America and Europe. We selected 23 loci containing repeat number polymorphisms. Using these primer sets, we conducted fragment analysis using the Terminator v3.1 cycle sequencing kit on a 3130xl Genetic analyzer (Life Technologies, Inc., Grand Island, NY). Allele sizes were determined using the LIZ 1200 size standard in GeneMapper version 4.0.

### Phylogenetics.

PAUP* version 4.0 a150 (66) was used to conduct maximum parsimony analysis on SNP data, and neighbor-joining analysis (based on mean character difference) on microsatellite data. Extended majority rule maximum likelihood phylogenetic trees were created from concatenated SNP sequences with RAxML version 8.2.7 (67) using the ASC_GTRGAMMA substitution model with ascertainment bias correction for each base character to account for invariant loci and the autoMRE bootstopping criterion with a maximum of 1,000 bootstraps. Trees were plotted with FigTree version 1.4.0 (A. Rambaut, 2012; http://tree.bio.ed.ac.uk/software/figtree/).

BEAST2 version 2.4.5 (68) was used to create maximum clade credibility trees from SNP locus data. Briefly, the SNP data were first filtered to remove SNPs within 50 bp of each other to minimize the effects of linkage on the analyses. Optimum base substitution models for SNP data were determined with the R package phangorn version 2.1.1 (69), resulting in the generalized time-reversible (GTR) model for the full analysis and the JC69 model for the North American isolates alone. Both analyses used a strict molecular clock and a coalescent constant population tree model. Skyline demographic models, which may be more appropriate for the expanding populations in North America as well as a relaxed log normal molecular clock, were attempted for comparison but failed to coalesce. Because only core SNPs were being analyzed, the analyses were corrected for ascertainment bias with a count of invariant bases in the nonduplicated alignments to the reference genome. The Markov chain Monte Carlo (MCMC) chain length was set to 10 million, with 10% burn-in and logging every 1,000 steps. Five chains were run, producing a total of 50,000 trees, which were combined and subsampled to 10,000 trees with LogCombiner version 2.4.3 and used to create an annotated maximum clade credibility tree with TreeAnnotator version 2.4.3.

### Population genetics.

Simpson’s index of genetic diversity and index of association were calculated with the R package poppr version 2.3.0 (70) as follows. SNP allele information was preprocessed by determining genetic distances between individuals with bitwise.dist, calculating the threshold distance for clustering isolates with cutoff_predictor, and assigning isolates to genotypes with mlg.filter. Simpson’s index of genetic diversity was determined for both SNP and microsatellite genotypes with diversity_ci with 1,000,000 bootstraps. North American isolates were rarefied to equal the number of European isolates in the SNP analysis, which enabled the comparison of estimated Simpson’s indices between the populations but prevented the calculation of standard error for the European population estimate. Bootstrapping with 1,000 samples to determine the mean standard index of association from SNP allele data was conducted with samp.ia, whereas the standard index of association and one-sided permutation test were calculated directly for microsatellite genotypes with ia.

Bruvo’s genetic distance (which incorporates the number of repeats at a locus rather than just differences) for North American microsatellite genotypes was determined with poppr’s bruvo.dist function. A pairwise geographic distance matrix between sampling locations was created with the R package geosphere v.1.5-5 (71). The Mantel test was conducted with the R package ade4 v.1.7-5 (72). Significance for this test was determined with 99 replicates of a Monte Carlo permutation test.

### Accession number(s).

Accession numbers for the sequences of the isolates examined in this study are listed in Tables 1 and 2.

## References

[1] Fisher MC, Henk DA, Briggs CJ, Brownstein JS, Madoff LC, McCraw SL, Gurr SJ 2012 Emerging fungal threats to animal, plant and ecosystem health. Nature 484:186–194. doi:10.1038/nature10947. 22498624 PMC3821985

[2] Ellison AM, Bank MS, Clinton BD, Colburn EA, Elliott K, Ford CR, Foster DR, Kloeppel BD, Knoepp JD, Lovett GM, Mohan J, Orwig DA, Rodenhouse NL, Sobczak WV, Stinson KA, Stone JK, Swan CM, Thompson J, Von Holle B, Webster JR 2005 Loss of foundation species: consequences for the structure and dynamics of forested ecosystems. Front Ecol Environ 3:479–486. doi:10.1890/1540-9295(2005)003[0479:LOFSCF]2.0.CO;2.

[3] Loo JA 2009 Ecological impacts of non-indigenous invasive fungi as forest pathogens. Biol Invasions 11:81–96. doi:10.1007/s10530-008-9321-3.

[4] Berger L, Speare R, Daszak P, Green DE, Cunningham AA, Goggin CL, Slocombe R, Ragan MA, Hyatt AD, McDonald KR, Hines HB, Lips KR, Marantelli G, Parkes H 1998 Chytridiomycosis causes amphibian mortality associated with population declines in the rain forests of Australia and Central America. Proc Natl Acad Sci U S A 95:9031–9036. doi:10.1073/pnas.95.15.9031. 9671799 PMC21197

[5] Bohuski E, Lorch JM, Griffin KM, Blehert DS 2015 TaqMan real-time polymerase chain reaction for detection of *Ophidiomyces ophiodiicola*, the fungus associated with snake fungal disease. BMC Vet Res 11:95. doi:10.1186/s12917-015-0407-8. 25889462 PMC4404600

[6] Stegen G, Pasmans F, Schmidt BR, Rouffaer LO, Van Praet S, Schaub M, Canessa S, Laudelout A, Kinet T, Adriaensen C, Haesebrouck F, Bert W, Bossuyt F, Martel A 2017 Drivers of salamander extirpation mediated by *Batrachochytrium salamandrivorans*. Nature 544:353–356. doi:10.1038/nature22059. 28425998

[7] Skerratt LF, Berger L, Speare R, Cashins S, McDonald KR, Phillott AD, Hines HB, Kenyon N 2007 Spread of chytridiomycosis has caused the rapid global decline and extinction of frogs. Ecohealth 4:125–134. doi:10.1007/s10393-007-0093-5.

[8] Blehert DS, Hicks AC, Behr M, Meteyer CU, Berlowski-Zier BM, Buckles EL, Coleman JT, Darling SR, Gargas A, Niver R, Okoniewski JC, Rudd RJ, Stone WB 2009 Bat white-nose syndrome: an emerging fungal pathogen? Science 323:227. doi:10.1126/science.1163874. 18974316

[9] Frick WF, Puechmaille SJ, Hoyt JR, Nickel BA, Langwig KE, Foster JT, Barlow KE, Bartonička T, Feller D, Haarsma A-J, Herzog C, Horáček I, van der Kooij J, Mulkens B, Petrov B, Reynolds R, Rodrigues L, Stihler CW, Turner GG, Kilpatrick AM 2015 Disease alters macroecological patterns of North American bats. Glob Ecol Biogeogr 24:741–749. doi:10.1111/geb.12290.

[10] Langwig KE, Frick WF, Reynolds R, Parise KL, Drees KP, Hoyt JR, Cheng TL, Kunz TH, Foster JT, Kilpatrick AM 2015 Host and pathogen ecology drive the seasonal dynamics of a fungal disease, white-nose syndrome. Proc Biol Sci 282:20142335. doi:10.1098/rspb.2014.2335. PMC4286034 25473016

[11] Frick WF, Pollock JF, Hicks AC, Langwig KE, Reynolds DS, Turner GG, Butchkoski CM, Kunz TH 2010 An emerging disease causes regional population collapse of a common North American bat species. Science 329:679–682. doi:10.1126/science.1188594. 20689016

[12] Langwig KE, Frick WF, Bried JT, Hicks AC, Kunz TH, Kilpatrick AM 2012 Sociality, density-dependence and microclimates determine the persistence of populations suffering from a novel fungal disease, white-nose syndrome. Ecol Lett 15:1050–1057. doi:10.1111/j.1461-0248.2012.01829.x. 22747672

[13] Kunz TH, Braun de Torrez E, Bauer D, Lobova T, Fleming TH 2011 Ecosystem services provided by bats. Ann N Y Acad Sci 1223:1–38. doi:10.1111/j.1749-6632.2011.06004.x. 21449963

[14] Rachowicz LJ, Hero J-M, Alford RA, Taylor JW, Morgan JAT, Vredenburg VT, Collins JP, Briggs CJ 2005 The novel and endemic pathogen hypotheses: competing explanations for the origin of emerging infectious diseases of wildlife. Conserv Biol 19:1441–1448. doi:10.1111/j.1523-1739.2005.00255.x.

[15] Kilpatrick AM, Briggs CJ, Daszak P 2010 The ecology and impact of chytridiomycosis: an emerging disease of amphibians. Trends Ecol Evol 25:109–118. doi:10.1016/j.tree.2009.07.011. 19836101

[16] Tibayrenc M, Ayala FJ 2012 Reproductive clonality of pathogens: a perspective on pathogenic viruses, bacteria, fungi, and parasitic protozoa. Proc Natl Acad Sci U S A 109:E3305–E3313. doi:10.1073/pnas.1212452109. PMC3511763 22949662

[17] Chaturvedi V, Springer DJ, Behr MJ, Ramani R, Li X, Peck MK, Ren P, Bopp DJ, Wood B, Samsonoff WA, Butchkoski CM, Hicks AC, Stone WB, Rudd RJ, Chaturvedi S 2010 Morphological and molecular characterizations of psychrophilic fungus *Geomyces destructans* from New York bats with white nose syndrome (WNS). PLoS One 5:e10783. doi:10.1371/journal.pone.0010783. 20520731 PMC2875398

[18] Meteyer CU, Buckles EL, Blehert DS, Hicks AC, Green DE, Shearn-Bochsler V, Thomas NJ, Gargas A, Behr MJ 2009 Histopathologic criteria to confirm white-nose syndrome in bats. J Vet Diagn Invest 21:411–414. doi:10.1177/104063870902100401. 19564488

[19] Palmer JM, Kubatova A, Novakova A, Minnis AM, Kolarik M, Lindner DL 2014 Molecular characterization of a heterothallic mating system in *Pseudogymnoascus destructans*, the fungus causing white-nose syndrome of bats. G3 4:1755–1763. doi:10.1534/g3.114.012641. 25053709 PMC4169168

[20] Khankhet J, Vanderwolf KJ, McAlpine DF, McBurney S, Overy DP, Slavic D, Xu J 2014 Clonal expansion of the *Pseudogymnoascus destructans* genotype in North America is accompanied by significant variation in phenotypic expression. PLoS One 9:e104684. doi:10.1371/journal.pone.0104684. 25122221 PMC4133243

[21] Ren P, Haman KH, Last LA, Rajkumar SS, Keel MK, Chaturvedi V 2012 Clonal spread of *Geomyces destructans* among bats, midwestern and southern United States. Emerg Infect Dis 18:883–885. doi:10.3201/eid1805.111711. 22516471 PMC3358064

[22] Pearson T, Okinaka RT, Foster JT, Keim P 2009 Phylogenetic understanding of clonal populations in an era of whole genome sequencing. Infect Genet Evol 9:1010–1019. doi:10.1016/j.meegid.2009.05.014. 19477301

[23] Van Belkum A 2007 Tracing isolates of bacterial species by multilocus variable number of tandem repeat analysis (MLVA). FEMS Immunol Med Microbiol 49:22–27. doi:10.1111/j.1574-695X.2006.00173.x. 17266711

[24] Etienne KA, Gillece J, Hilsabeck R, Schupp JM, Colman R, Lockhart SR, Gade L, Thompson EH, Sutton DA, Neblett-Fanfair R, Park BJ, Turabelidze G, Keim P, Brandt ME, Deak E, Engelthaler DM 2012 Whole genome sequence typing to investigate the *Apophysomyces* outbreak following a tornado in Joplin, Missouri, 2011. PLoS One 7:e49989. doi:10.1371/journal.pone.0049989. 23209631 PMC3507928

[25] Hoyt JR, Sun K, Parise KL, Lu G, Langwig KE, Jiang TT, Yang S, Frick WF, Kilpatrick AM, Foster JT, Feng J 2016 Widespread bat white-nose syndrome fungus, northeastern China. Emerg Infect Dis 22:140–142. doi:10.3201/eid2201.151314. 26673906 PMC4698868

[26] Drees KP, Palmer JM, Sebra R, Lorch JM, Chen C, Wu CC, Bok JW, Keller NP, Blehert DS, Cuomo CA, Lindner DL, Foster JT 2016 Use of multiple sequencing technologies to produce a high-quality genome of the fungus *Pseudogymnoascus destructans*, the causative agent of bat white-nose syndrome. Genome Announc 4:e00445-16. doi:10.1128/genomeA.00445-16. 27365344 PMC4929507

[27] Minnis AM, Lindner DL 2013 Phylogenetic evaluation of *Geomyces* and allies reveals no close relatives of *Pseudogymnoascus destructans*, comb. nov., in bat hibernacula of eastern North America. Fungal Biol 117:638–649. doi:10.1016/j.funbio.2013.07.001. 24012303

[28] Leopardi S, Blake D, Puechmaille SJ 2015 White-nose syndrome fungus introduced from Europe to North America. Curr Biol 25:R217–R219. doi:10.1016/j.cub.2015.01.047. 25784035

[29] Agapow P-M, Burt A 2001 Indices of multilocus linkage disequilibrium. Mol Ecol Notes 1:101–102. doi:10.1046/j.1471-8278.2000.00014.x.

[30] Puechmaille SJ 2016 The program Structure does not reliably recover the correct population structure when sampling is uneven: subsampling and new estimators alleviate the problem. Mol Ecol Resour 16:608–627. doi:10.1111/1755-0998.12512. 26856252

[31] Rajkumar SS, Li X, Rudd RJ, Okoniewski JC, Xu J, Chaturvedi S, Chaturvedi V 2011 Clonal genotype of *Geomyces destructans* among bats with white nose syndrome, New York, USA. Emerg Infect Dis 17:1273–1276. doi:10.3201/eid1707.102056. 21762585 PMC3381392

[32] Trivedi J, Lachapelle J, Vanderwolf KJ, Misra V, Willis CKR, Ratcliffe JM, Ness RW, Anderson JB, Kohn LM 2017 Fungus causing white-nose syndrome in bats accumulates genetic variability in North America with no sign of recombination. mSphere 2:e00271-17. doi:10.1128/mSphereDirect.00271-17. 28713859 PMC5506559

[33] Fontaine MC, Austerlitz F, Giraud T, Labbé F, Papura D, Richard-Cervera S, Delmotte F 2013 Genetic signature of a range expansion and leap-frog event after the recent invasion of Europe by the grapevine downy mildew pathogen *Plasmopara viticola*. Mol Ecol 22:2771–2786. doi:10.1111/mec.12293. 23506060

[34] Gladieux P, Feurtey A, Hood ME, Snirc A, Clavel J, Dutech C, Roy M, Giraud T 2015 The population biology of fungal invasions. Mol Ecol 24:1969–1986. doi:10.1111/mec.13028. 25469955

[35] Eppinger M, Pearson T, Koenig SS, Pearson O, Hicks N, Agrawal S, Sanjar F, Galens K, Daugherty S, Crabtree J, Hendriksen RS, Price LB, Upadhyay BP, Shakya G, Fraser CM, Ravel J, Keim PS 2014 Genomic epidemiology of the Haitian cholera outbreak: a single introduction followed by rapid, extensive, and continued spread characterized the onset of the epidemic. mBio 5:e01721. doi:10.1128/mBio.01721-14. 25370488 PMC4222100

[36] Frost SD, Volz EM 2013 Modelling tree shape and structure in viral phylodynamics. Philos Trans R Soc Lond B Biol Sci 368:20120208. doi:10.1098/rstb.2012.0208. 23382430 PMC3678332

[37] Poon AFY, Walker LW, Murray H, McCloskey RM, Harrigan PR, Liang RH 2013 Mapping the shapes of phylogenetic trees from human and zoonotic RNA viruses. PLoS One 8:e78122. doi:10.1371/journal.pone.0078122. 24223766 PMC3815201

[38] Dutech C, Barrès B, Bridier J, Robin C, Milgroom MG, Ravigné V 2012 The chestnut blight fungus world tour: successive introduction events from diverse origins in an invasive plant fungal pathogen. Mol Ecol 21:3931–3946. doi:10.1111/j.1365-294X.2012.05575.x. 22548317

[39] Zukal J, Bandouchova H, Brichta J, Cmokova A, Jaron KS, Kolarik M, Kovacova V, Kubátová A, Nováková A, Orlov O, Pikula J, Presetnik P, Šuba J, Zahradníková A Jr, Martínková N 2016 White-nose syndrome without borders: *Pseudogymnoascus destructans* infection tolerated in Europe and Palearctic Asia but not in North America. Sci Rep 6:19829. doi:10.1038/srep19829. 26821755 PMC4731777

[40] Tibayrenc M, Kjellberg F, Arnaud J, Oury B, Brenière SF, Dardé ML, Ayala FJ 1991 Are eukaryotic microorganisms clonal or sexual? A population genetics vantage. Proc Natl Acad Sci U S A 88:5129–5133. doi:10.1073/pnas.88.12.5129. 1675793 PMC51825

[41] Warnecke L, Turner JM, Bollinger TK, Lorch JM, Misra V, Cryan PM, Wibbelt G, Blehert DS, Willis CKR 2012 Inoculation of bats with European *Geomyces destructans* supports the novel pathogen hypothesis for the origin of white-nose syndrome. Proc Natl Acad Sci U S A 109:6999–7003. doi:10.1073/pnas.1200374109. 22493237 PMC3344949

[42] Puechmaille SJ, Verdeyroux P, Fuller H, Gouilh MA, Bekaert M, Teeling EC 2010 White-nose syndrome fungus (Geomyces destructans) in bat, France. Emerg Infect Dis 16:290–293. doi:10.3201/eid1602.091391. 20113562 PMC2958029

[43] Wibbelt G, Kurth A, Hellmann D, Weishaar M, Barlow A, Veith M, Prüger J, Görföl T, Grosche L, Bontadina F, Zöphel U, Seidl HP, Seidl HP, Blehert DS 2010 White-nose syndrome fungus (*Geomyces destructans*) in bats, Europe. Emerg Infect Dis 16:1237–1243. doi:10.3201/eid1608.100002. 20678317 PMC3298319

[44] Barlow AM, Worledge L, Miller H, Drees KP, Wright P, Foster JT, Sobek C, Borman AM, Fraser M 2015 First confirmation of *Pseudogymnoascus destructans* in British bats and hibernacula. Vet Rec 177:73. doi:10.1136/vr.102923. 25968064

[45] Paiva-Cardoso MdN, Morinha F, Barros P, Vale-Gonçalves H, Coelho AC, Fernandes L, Travassos P, Faria AS, Bastos E, Santos M, Cabral JA 2014 First isolation of *Pseudogymnoascus destructans* in bats from Portugal. Eur J Wildl Res 60:645–649. doi:10.1007/s10344-014-0831-2.

[46] Pavlinić I, Đaković M, Lojkić I 2015 Pseudogymnoascus destructans in Croatia confirmed. Eur J Wildl Res 61:325–328. doi:10.1007/s10344-014-0885-1.

[47] Bandouchova H, Bartonicka T, Berkova H, Brichta J, Cerny J, Kovacova V, Kolarik M, Köllner B, Kulich P, Martínková N, Rehak Z, Turner GG, Zukal J, Pikula J 2015 *Pseudogymnoascus destructans*: evidence of virulent skin invasion for bats under natural conditions, Europe. Transbound Emerg Dis 62:1–5. doi:10.1111/tbed.12282. 25268034

[48] Pikula J, Bandouchova H, Novotny L, Meteyer CU, Zukal J, Irwin NR, Zima J, Martínková N 2012 Histopathology confirms white-nose syndrome in bats in Europe. J Wildl Dis 48:207–211. doi:10.7589/0090-3558-48.1.207. 22247393

[49] Puechmaille SJ, Wibbelt G, Korn V, Fuller H, Forget F, Mühldorfer K, Kurth A, Bogdanowicz W, Borel C, Bosch T, Cherezy T, Drebet M, Görföl T, Haarsma AJ, Herhaus F, Hallart G, Hammer M, Jungmann C, Le Bris Y, Lutsar L, Masing M, Mulkens B, Passior K, Starrach M, Wojtaszewski A, Zöphel U, Teeling EC 2011 Pan-European distribution of white-nose syndrome fungus (*Geomyces destructans*) not associated with mass mortality. PLoS One 6:e19167. doi:10.1371/journal.pone.0019167. 21556356 PMC3083413

[50] Puechmaille SJ, Frick WF, Kunz TH, Racey PA, Voigt CC, Wibbelt G, Teeling EC 2011 White-nose syndrome: is this emerging disease a threat to European bats? Trends Ecol Evol 26:570–576. doi:10.1016/j.tree.2011.06.013. 21835492

[51] Rosenblum EB, James TY, Zamudio KR, Poorten TJ, Ilut D, Rodriguez D, Eastman JM, Richards-Hrdlicka K, Joneson S, Jenkinson TS, Longcore JE, Parra Olea G, Toledo LF, Arellano ML, Medina EM, Restrepo S, Flechas SV, Berger L, Briggs CJ, Stajich JE 2013 Complex history of the amphibian-killing chytrid fungus revealed with genome resequencing data. Proc Natl Acad Sci U S A 110:9385–9390. doi:10.1073/pnas.1300130110. 23650365 PMC3677446

[52] Knaus BJ, Tabima JF, Davis CE, Judelson HS, Grünwald NJ 2016 Genomic analyses of dominant U.S. clonal lineages of *Phytophthora infestans* reveals a shared common ancestry for clonal lineages US11 and US18 and a lack of recently shared ancestry among all other U.S. lineages. Phytopathology 106:1393–1403. doi:10.1094/PHYTO-10-15-0279-R. 27348344

[53] Gargas A, Trest MT, Christensen M, Volk TJ, Blehert DS 2009 *Geomyces destructans* sp. nov. associated with bat white-nose syndrome. Mycotaxon 108:147–154. doi:10.5248/108.147.

[54] Shuey MM, Drees KP, Lindner DL, Keim P, Foster JT 2014 Highly sensitive quantitative PCR for the detection and differentiation of *Pseudogymnoascus destructans* and other *Pseudogymnoascus* species. Appl Environ Microbiol 80:1726–1731. doi:10.1128/AEM.02897-13. 24375140 PMC3957615

[55] Gillece JD, Schupp JM, Balajee SA, Harris J, Pearson T, Yan Y, Keim P, DeBess E, Marsden-Haug N, Wohrle R, Engelthaler DM, Lockhart SR 2011 Whole genome sequence analysis of *Cryptococcus gattii* from the Pacific Northwest reveals unexpected diversity. PLoS One 6:e28550. doi:10.1371/journal.pone.0028550. 22163313 PMC3233577

[56] Kozarewa I, Turner DJ 2011 96-plex molecular barcoding for the Illumina genome analyzer, p 279–298. Humana Press, New York, NY. doi:10.1007/978-1-61779-089-8_20. 21431778

[57] Kapa Biosystems 2012 High-throughput NGS library preparation technical guide, Illumina platforms, KR0427, 1 12 Kapa Biosystems, Wilmington, MA.

[58] Sahl JW, Lemmer D, Travis J, Schupp JM, Gillece JD, Aziz M, Driebe EM, Drees KP, Hicks ND, Williamson CHD, Hepp CM, Smith DE, Roe C, Engelthaler DM, Wagner DM, Keim P 2016 NASP: an accurate, rapid method for the identification of SNPs in WGS datasets that supports flexible input and output formats. Microbial Genomics 2:e000074. doi:10.1099/mgen.0.000074. 28348869 PMC5320593

[59] Li H, Durbin R 2010 Fast and accurate long-read alignment with Burrows-Wheeler transform. Bioinformatics 26:589–595. doi:10.1093/bioinformatics/btp698. 20080505 PMC2828108

[60] McKenna A, Hanna M, Banks E, Sivachenko A, Cibulskis K, Kernytsky A, Garimella K, Altshuler D, Gabriel S, Daly M, DePristo MA 2010 The Genome Analysis Toolkit: a MapReduce framework for analyzing next-generation DNA sequencing data. Genome Res 20:1297–1303. doi:10.1101/gr.107524.110. 20644199 PMC2928508

[61] Van der Auwera GA, Carneiro MO, Hartl C, Poplin R, del Angel G, Levy-Moonshine A, Jordan T, Shakir K, Roazen D, Thibault J, Banks E, Garimella KV, Altshuler D, Gabriel S, DePristo MA 2013 From FastQ data to high-confidence variant calls: the Genome Analysis Toolkit best practices pipeline. Curr Protoc Bioinformatics 43:11.10.1–11.10.33. doi:10.1002/0471250953.bi1110s43. 25431634 PMC4243306

[62] DePristo MA, Banks E, Poplin R, Garimella KV, Maguire JR, Hartl C, Philippakis AA, del Angel G, Rivas MA, Hanna M, McKenna A, Fennell TJ, Kernytsky AM, Sivachenko AY, Cibulskis K, Gabriel SB, Altshuler D, Daly MJ 2011 A framework for variation discovery and genotyping using next-generation DNA sequencing data. Nat Genet 43:491–498. doi:10.1038/ng.806. 21478889 PMC3083463

[63] Kurtz S, Phillippy A, Delcher AL, Smoot M, Shumway M, Antonescu C, Salzberg SL 2004 Versatile and open software for comparing large genomes. Genome Biol 5:R12. doi:10.1186/gb-2004-5-2-r12. 14759262 PMC395750

[64] Cingolani P, Platts A, Wang LL, Coon M, Nguyen T, Wang L, Land SJ, Lu X, Ruden DM 2012 A program for annotating and predicting the effects of single nucleotide polymorphisms, SnpEff: SNPs in the genome of Drosophila melanogaster strain w1118; iso-2; iso-3. Fly 6:80–92. doi:10.4161/fly.19695. 22728672 PMC3679285

[65] Drees KP, Parise KL, Rivas SM, Felton LL, Puechmaille SJ, Keim P, Foster JT 2017 Characterization of microsatellites in *Pseudogymnoascus destructans* for white-nose syndrome genetic analysis. J Wildl Dis 53:869–874. doi:10.7589/2016-09-217. 28475452

[66] Swofford DL 2003 PAUP*: phylogenetic analysis using parsimony (* and other methods), version 4.0 b10 Sinauer Associates, Sunderland, MA.

[67] Stamatakis A, Blagojevic F, Nikolopoulos DS, Antonopoulos CD 2007 Exploring new search algorithms and hardware for phylogenetics: RAxML meets the IBM cell. J VLSI Signal Process Syst Signal Image Video Technol 48:271–286. doi:10.1007/s11265-007-0067-4.

[68] Drummond AJ, Rambaut A 2007 BEAST: Bayesian evolutionary analysis by sampling trees. BMC Evol Biol 7:214. doi:10.1186/1471-2148-7-214. 17996036 PMC2247476

[69] Schliep KP 2011 phangorn: phylogenetic analysis in R. Bioinformatics 27:592–593. doi:10.1093/bioinformatics/btq706. 21169378 PMC3035803

[70] Kamvar ZN, Brooks JC, Grünwald NJ 2015 Novel R tools for analysis of genome-wide population genetic data with emphasis on clonality. Front Genet 6:208. doi:10.3389/fgene.2015.00208. 26113860 PMC4462096

[71] Karney CFF 2013 Algorithms for geodesics. J Geodesy 87:43–55. doi:10.1007/s00190-012-0578-z.

[72] Chessel D, Dufour AB, Thioulouse J 2004 The ade4 package—I: one-table methods. R News 4:5–10.

[73] Palmer JM, Drees KP, Foster JT, Lindner DL Extreme sensitivity to ultra-violet light in the fungal pathogen causing white-nose syndrome of bats. Nature Communications, in press. 10.1038/s41467-017-02441-z PMC5750222 29295979

